# 3D Printing of Stretchable, Adhesive and Conductive Ti_3_C_2_T_x_-Polyacrylic Acid Hydrogels

**DOI:** 10.3390/polym14101992

**Published:** 2022-05-13

**Authors:** Weijing Zhao, Jie Cao, Fucheng Wang, Fajuan Tian, Wenqian Zheng, Yuqian Bao, Kaiyue Zhang, Zhilin Zhang, Jiawen Yu, Jingkun Xu, Ximei Liu, Baoyang Lu

**Affiliations:** 1 Department of Endocrinology and Metabolism, Shanghai Jiao Tong University Affiliated Sixth People’s Hospital, Shanghai Clinical Center for Diabetes, Shanghai Key Clinical Center for Metabolic Disease, Shanghai Diabetes Institute, Shanghai Key Laboratory of Diabetes Mellitus, Shanghai Jiao Tong University, Shanghai 200240, China; z811wj@163.com (W.Z.); yqbao@sjtu.edu.cn (Y.B.); 2 Flexible Electronics Innovation Institute, Jiangxi Key Laboratory of Flexible Electronics, Jiangxi Science & Technology Normal University, Nanchang 330013, China; caojie9905@163.com (J.C.); wfc317500626@163.com (F.W.); fajuantian2020@163.com (F.T.); cathy9806@163.com (W.Z.); zky193722@163.com (K.Z.); zhangzhilin1117@163.com (Z.Z.); yujw0629@163.com (J.Y.); xujingkun@tsinghua.org.cn (J.X.); 3 School of Chemistry & Chemical Engineering, Jiangxi Science & Technology Normal University, Nanchang 330013, China; 4 School of Pharmacy, Jiangxi Science & Technology Normal University, Nanchang 330013, China

**Keywords:** MXene, conductive hydrogel, 3D printing, pre-crosslinking, adhesion

## Abstract

Stretchable, adhesive, and conductive hydrogels have been regarded as ideal interfacial materials for seamless and biocompatible integration with the human body. However, existing hydrogels can rarely achieve good mechanical, electrical, and adhesive properties simultaneously, as well as limited patterning/manufacturing techniques posing severe challenges to bioelectronic research and their practical applications. Herein, we develop a stretchable, adhesive, and conductive Ti_3_C_2_T_x_-polyacrylic acid hydrogel by a simple pre-crosslinking method followed by successive direct ink writing 3D printing. Pre-polymerization of acrylic acid can be initiated by mechanical mixing with Ti_3_C_2_T_x_ nanosheet suspension, leading to the formation of viscous 3D printable ink. Secondary free radical polymerization of the ink patterns via 3D printing can achieve a stretchable, adhesive, and conductive Ti_3_C_2_T_x_-polyacrylic acid hydrogel. The as-formed hydrogel exhibits remarkable stretchability (~622%), high electrical conductivity (5.13 S m^−1^), and good adhesion strength on varying substrates. We further demonstrate the capability of facilely printing such hydrogels into complex geometries like mesh and rhombus patterns with high resolution and robust integration.

## 1. Introduction

Bioelectronic interfacing with the human body has become a bridge to record/stimulate physiological information in our daily lives. Currently, most bioelectronic devices are fabricated from inorganic materials with proper electrical conductivity, such as metals and silicon [1,2,3]. However, these bioelectronic devices based on inorganic materials are limited by mechanical mismatches with biological tissues, leading to unstable and uncomfortable signal collection/stimulation [4,5,6]. Compared to traditional inorganic materials, tissue-like soft materials can replace traditional rigid electronics to improve compliance and improve performance to suffice for practical applications [3,6,7,8,9].

Hydrogels have been extensively explored in bioelectronics because of their tissue-like mechanical properties and tunable functionalities. Most recently, an increasing number of functional hydrogels have been employed for the fabrication/integration of bioelectronic devices [6,7,10]. Among them, stretchable, adhesive, and conductive hydrogels have been regarded as ideal candidates in human-machine interfaces [11]. However, the development of such hydrogels is still a considerable challenge due to the trade-offs among varying properties. 

One of the effective strategies to realize stretchable, adhesive, and conductive hydrogels is rational compositing of highly conductive nanofillers within the hydrogel matrix. MXene is an emerging family of 2D materials with a general formula of M_n+1_X_n_T_x_, which are obtained by selective etching A-group (generally group IIIA and IVA elements) layers from the MAX phases [12,13,14]. Typically, the “M” is a transition metal, “X” is carbon and/or nitrogen, and “T” represents the surface functional groups (=O, -OH, and -F). Particularly, Ti_3_C_2_T_x_, as a representative MXene material, possesses high electrical conductivity (10,000 S cm^−1^), superior hydrophilicity [15,16], as well as good mechanical performance due to the presence of M-N or M-C bonds. Functional groups (=O, -OH, and -F) on the MXene surface [17] enables MXenes with excellent dispersibility, which can be processed by multiple manufacturing techniques [18,19,20], involving screen-printing [21,22], stamping [23], and spraying [24]. However, these processing techniques are usually limited by multiple factors, such as low-resolution, two-dimensional, and low aspect ratio [25]. Direct ink writing 3D printing is an advanced additive manufacturing technology that offers the capability to fabricate geometrically freeform 3D structures [26,27]. Although some interesting efforts have been devoted to develop Mxene-based printable inks [28,29,30], 3D printing of Mxene-based hydrogels simultaneously with good mechanical, electrical, and adhesive properties have been rarely investigated.

In this work, we prepare a stretchable, conductive, and adhesive Ti_3_C_2_T_x_-polyacrylic acid (PAA) hydrogel by using a simple pre-crosslinking method followed by direct ink writing 3D printing. Our strategy is to employ Ti_3_C_2_T_x_ nanosheet aqueous suspension to initiate the pre-polymerization of acrylic acid monomers. Ti_3_C_2_T_x_-PAA hydrogels can be further achieved by secondary radical polymerization of 3D printed ink patterns. The resultant Ti_3_C_2_T_x_-PAA hydrogel exhibits high stretchability (~622%), high electrical conductivity (5.13 S m^−1^), and strong adhesion on varying substrates. The flexibility of 3D printing technology enables facile patterning of complex geometries like mesh and rhombus patterns with high resolution and robust integration. Based on these merits, Ti_3_C_2_T_x_-PAA hydrogels are potential material candidates for biomedical applications [30,31,32].

## 2. Materials and Methods

### 2.1. Materials

Acrylic acid (AA, 99%; Shanghai Vita, Shanghai, China), ammonium persulfate (APS, ≥98%; Aladdin, Shanghai, China), lithium fluoride (LiF, 97%; J&K Scientific, Beijing, China), hydrochloric acid (HCl, 35–38%; Shanghai Vita, Shanghai, China), phosphate buffer saline (PBS, pH = 7.4; Howei Pharm, Guangzhou, China)), and Ti_3_AlC_2_ were purchased from 11 Technolog Co, Jilin, China.

*Preparation of Ti_3_C_2_T_x_ MXene nanosheets.* MXene was prepared by a selective etching method with LiF/HCl as the etching solution. Notably, the etching solution was composed of LiF (1 g) and HCl (20 mL, 9 M). After the etching solution was stirred for 5 min at room temperature, Ti_3_AlC_2_ powder (1 g) was slowly added to the etching solution and stirred at 35 °C for 24 h. When the reaction was completed, the acid suspension was repeatedly washed with deionized water 6 times and centrifuged at 6000 rpm for 2 min. Deionized water was added to the collected sediment, followed by the suspension being sonicated for 1 h and centrifuged at 2000 rpm for 30 min. Subsequently, a dark green MXene colloidal dispersion was collected [33]. Redispersible MXene powder can be obtained by drying MXene colloidal dispersion at 60 °C for 6 h. Such powder (0.1 g) was added to deionized water (1.33 g) and sonicated for 30 min to obtain aqueous MXene nanosheet suspension.

*Preparation of 3D printable Ti_3_C_2_T_x_-PAA inks.* MXene suspension and AA monomers were mixed and filtered with a syringe filter (40 µm) to obtain viscous 3D printable Ti_3_C_2_T_x_-PAA inks via pre-crosslinking at the optimal time (Figure S1). The detailed composition of different Ti_3_C_2_T_x_-PAA inks is listed in Table S1.

*3D printing of Ti_3_C_2_T_x_-PAA inks*. Direct ink writing 3D printing of Ti_3_C_2_T_x_-PAA inks was conducted based on a 3D printer (DB 100, Shanghai Mifang Electronic Technology, Shanghai, China) with a 160-µm nozzle. The printing pressure and speed were 30 kPa and 40 mm s^−1^, respectively. Printing pattern paths were generated by AI drawings and converted into SVG. The detailed printing paths are shown in Figure S2. After printing, 3D-printed Ti_3_C_2_T_x_-PAA patterns were put into APS solution for 10 min to yield Ti_3_C_2_T_x_-PAA hydrogels via secondary radical polymerization of AA oligomer.

### 2.2. Characterization

*Mechanical characterization.* Mechanical properties of Ti_3_C_2_T_x_-PAA hydrogels were performed by using a universal testing machine (Zhiqu-990L, ZHIQU Precision Instrument, Guangzhou, China) equipped with a U-stretch 5 N load cell at 100 mm min^−1^ rate. 

*Electrical conductivity measurement.* Electrical conductivity of Ti_3_C_2_T_x_-PAA hydrogels was measured by using a standard four-point probe (Keithley 2700 digital multimeter, Keithley, Beaverton, OG, USA). Ti_3_C_2_T_x_-PAA hydrogels were cut into rectangle shapes (12 mm in length and 5 mm in width). Copper electrodes were adhered onto the surface of hydrogels by applying silver paste.

*Electrochemical properties.* Charge injection capacity (CIC) and electrochemical impedance spectroscopy (EIS) measurements of Ti_3_C_2_T_x_-PAA hydrogels were carried out by using a Gamry instrument (Interface 1010, Gamry instruments, Warminster, PA, USA). The samples were attached onto platinum substrate. All measurements were obtained using a three-electrode configuration, e.g., Ti_3_C_2_T_x_-PAA hydrogel as the working electrode, platinum wires as the counter electrode, and Ag/AgCl electrode as the reference electrode with PBS as the electrolyte.

*Adhesive properties.* The adhesion properties of Ti_3_C_2_T_x_-PAA hydrogels were evaluated through lap shear tests based on the ASTM F2255-05 standard by using a universal testing machine (ZQ-990LB, ZHIQU Precision Instrument, Dongguan, China) at the testing speed of 10 mm min^−1^. The adhesion strength was determined by dividing the maximum separation force by the contact area.

## 3. Results and Discussion

### 3.1. Design and Preparation of Ti_3_C_2_T_x_-PAA Hydrogels

**Figure 1:**
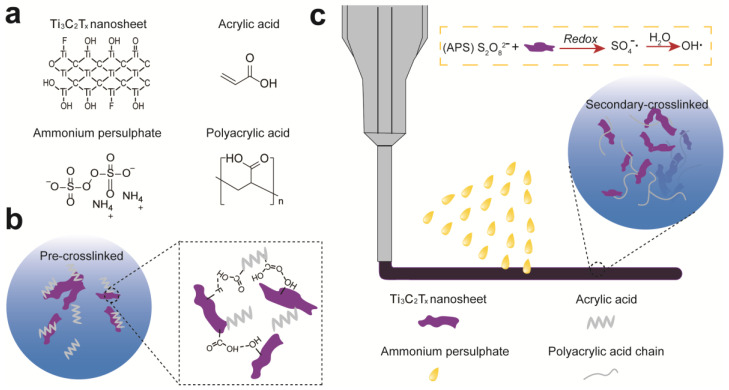
Schematic illustration for preparing Ti_3_C_2_T_x_-PAA hydrogels via 3D printing. (**a**) Chemical structures of Ti_3_C_2_T_x_ nanosheets, AA, APS, and PAA. (**b**) Pre-polymerization of AA with Ti_3_C_2_T_x_ nanosheets. (**c**) 3D printing and secondary-crosslinking of Ti_3_C_2_T_x_-PAA hydrogels with APS.

To prepare a stretchable, adhesive, and conductive hydrogel, we present a pre-crosslinking strategy to pre-polymerize AA monomer within Ti_3_C_2_T_x_ nanosheets (Figure 1a), leading to the viscosity increase and the formation of viscous 3D printable inks (Figure 1b). With increasing concentration of Ti_3_C_2_T_x_ nanosheet suspension, AA can polymerize gradually, resulting in the transition of mixed solution into viscous 3D printable pastes (Figure S1). This is mainly due to the catalytic effect of Ti_3_C_2_T_x_ and also hydrogen bonding between hydroxyl groups in Ti_3_C_2_T_x_ and carboxylic groups of AA or PAA chains [34].

After 3D printing, prepolymerized hydrogel patterns are further oxidized by putting them into APS solution for 10 min to obtain fully crosslinked Ti_3_C_2_T_x_-PAA hydrogels (Figure 1c). During the soaking process, a redox reaction between the reductive (Ti_3_C_2_T_x_ nanosheets) and the initiator (APS) results in plenty of sulfate radical (${\mathrm{SO}}_{4}^{-}\cdot$) generated from APS. Subsequently, ${\mathrm{SO}}_{4}^{-}\cdot$ hydrolyzes a large amount of hydroxyl radicals (⋅OH), which accelerates the polymerization of AA monomers or oligomers to form PAA chains [10,35]. Meanwhile, the redox reaction releases enormous heat, also facilitating the generation of free radicals for faster cross-linking [35,36].

### 3.2. Mechanical Performance of Ti_3_C_2_T_x_-PAA Hydrogels

**Figure 2:**
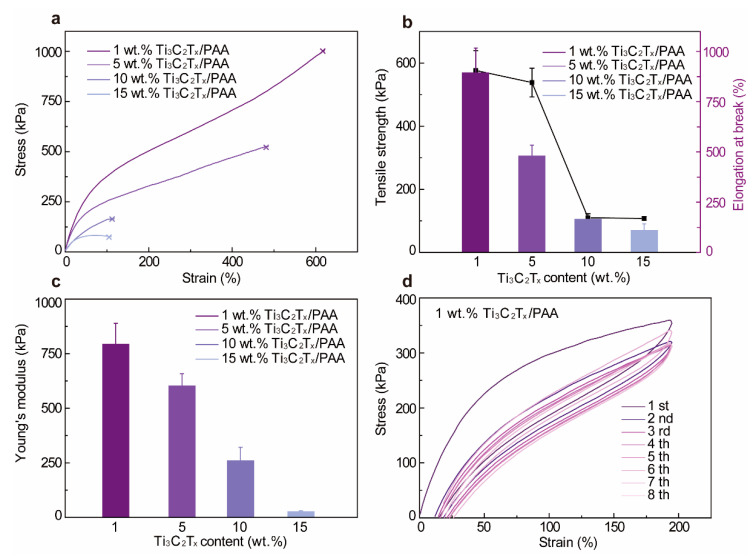
Mechanical properties of Ti_3_C_2_T_x_-PAA hydrogels. (**a**) Stress-strain curves, (**b**) tensile strength and elongation at the break, and (**c**) Young’s modulus with varying Ti_3_C_2_T_x_ contents. (**d**) Loading/unloading stress-strain curves at the strain of 200% for 1 wt.% Ti_3_C_2_T_x_-PAA hydrogel.

Since we prepare the hydrogel by directly adding AA monomers into the Ti_3_C_2_T_x_ dispersion, the water content of the resultant Ti_3_C_2_T_x_-PAA hydrogels is controlled by the Ti_3_C_2_T_x_ concentration, showing rising water content from 11.71% (1 wt.% Ti_3_C_2_T_x_) to 66.54% (15 wt.% Ti_3_C_2_T_x_) with increasing Ti_3_C_2_T_x_ concentration (Table S1). To quantify the mechanical properties of Ti_3_C_2_T_x_-PAA hydrogels, we systematically characterize the stress-strain curves of varying Ti_3_C_2_T_x_-PAA hydrogels by tensile tests. As shown in Figure 2, Ti_3_C_2_T_x_-PAA hydrogels display excellent mechanical characteristics with various Ti_3_C_2_T_x_ contents. Apparently, the incorporation of Ti_3_C_2_T_x_ dispersion dramatically decreases the ultimate strain from 622% to 101% and reduces the tensile strength from 893 kPa to 111 kPa (Figure 2a,b). This phenomenon can be ascribed to the introduction of rigid Ti_3_C_2_T_x_ nanosheets as well as the water content changes in Ti_3_C_2_T_x_-PAA hydrogels. The Young’s modulus of the Ti_3_C_2_T_x_-PAA hydrogels also declines from 795.8 kPa to 78 kPa (Figure 2c) with increasing Ti_3_C_2_T_x_ concentration, owing to the rising water content.

Most hydrogels require robust mechanical performance to resist various mechanical deformations. Therefore, we further evaluate the energy dissipation capacity of 1 wt.% Ti_3_C_2_T_x_-PAA hydrogel (Figure 2d). For consecutive tensile loading/unloading tests, the tensile stress decreases when increasing the cyclic time and maintains its elasticity (~88.55% of the original) after eight cycles. Evidently, the loading/unloading curve area gradually stabilizes from the second to the eighth lap, implying stable energy dissipation and further revealing the superior mechanical performance of Ti_3_C_2_T_x_-PAA hydrogels. Taking advantage of these merits, the resultant Ti_3_C_2_T_x_-PAA hydrogels exhibit favorable overall mechanical properties and can be easily tuned by varying the material composition.

### 3.3. Electrical and Electrochemical Properties of Ti_3_C_2_T_x_-PAA Hydrogels

**Figure 3:**
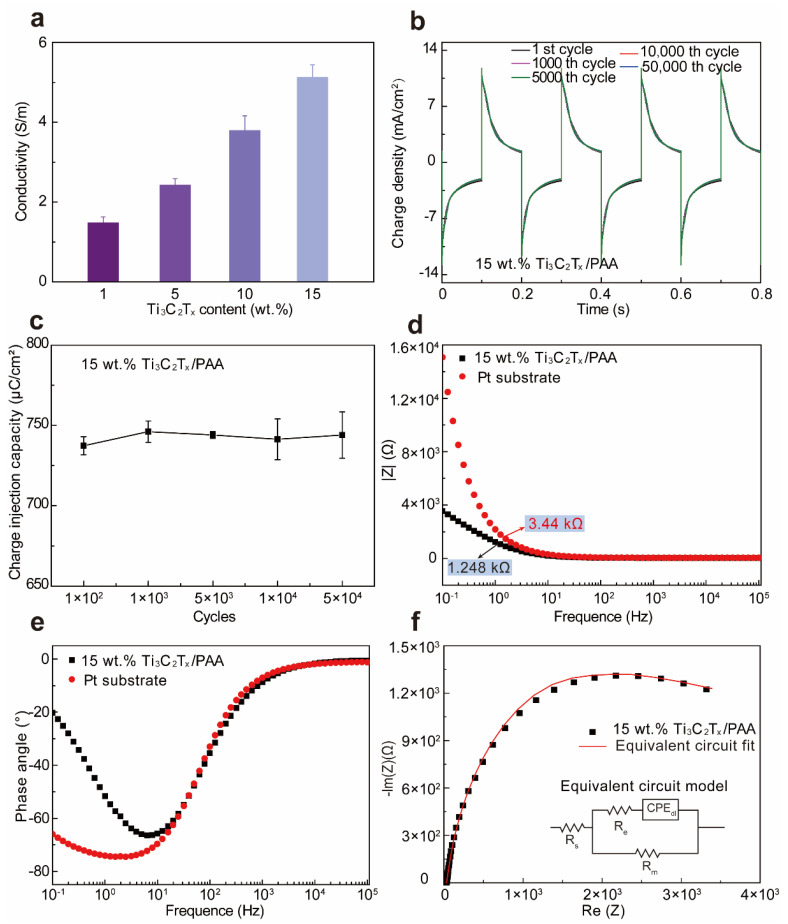
Electrical conductivity and electrochemical performances of Ti_3_C_2_T_x_-PAA hydrogels. (**a**) Electrical conductivity of Ti_3_C_2_T_x_-PAA hydrogels. (**b**) Cyclic current pulse injection curves of the 15 wt.% Ti_3_C_2_T_x_-PAA hydrogel on Pt electrode under between −1 V and 1 V (versus Ag/AgCl). (**c**) Charge injection capacity of the 15 wt.% Ti_3_C_2_T_x_-PAA hydrogel. From the EIS characterization (versus frequency of 0.1~10^5^ Hz), (**d**) plots of impedance, (**e**) phase angle, and (**f**) Nyquist plot of the 15 wt.% Ti_3_C_2_T_x_-PAA hydrogel on Pt substrate are obtained. The corresponding equivalent circuit fitted values of the 15 wt.% Ti_3_C_2_T_x_-PAA hydrogel are R_s_ = 32.47 Ω, R_m_ = 3678 Ω, R_e_ = 0.6955 Ω, and CPE_dl_ (Q_p_ = 1.479 × 10^−4^ S·s^n^, n_p_ = 0.8315) [4,25].

To assess the electrical conductivity of Ti_3_C_2_T_x_-PAA hydrogels, we vary the Ti_3_C_2_T_x_ concentration in the Ti_3_C_2_T_x_-PAA solution and keep the same soaking condition for 10 min in PBS solution. Evidently, when increasing the concentration of Ti_3_C_2_T_x_, the electrical conductivity of Ti_3_C_2_T_x_-PAA hydrogels is improved up to 5.13 S m^−1^ in PBS, displaying a positive linear relationship versus Ti_3_C_2_T_x_ concentration (Figure 3a) [37]. A significant increase in electrical conductivity is attributable to favorably connected Ti_3_C_2_T_x_ nanosheets for an extraordinary electron transport ability [37,38]. Notably, compared to other conductive hydrogels reported previously [39], our Ti_3_C_2_T_x_-PAA hydrogel exhibits superior conductivity to most hydrogels so far (see detailed comparison in Table S2 [36,40,41,42,43]). Highly conductive Ti_3_C_2_T_x_-PAA hydrogel may also be utilized for potential applications in flexible and wearable electronic devices [3,44].

To further investigate the electrochemical performance of Ti_3_C_2_T_x_-PAA hydrogels, we performed the current density of the 15 wt.% Ti_3_C_2_T_x_-PAA hydrogels. Under a biphasic voltage transient pulse test (±1 V voltage amplitude, 0.1 s duration), the 15 wt.% Ti_3_C_2_T_x_-PAA hydrogel displays the highest charge density of ~11.82 mA cm^−2^ (Figure 3b). The charge injection capacity (CIC) of Ti_3_C_2_T_x_-PAA hydrogel is measured to be about 742.6 ± 5 μC cm^−2^. Moreover, the CIC loss is less than 0.9% even after 50,000 cycles of bipolar voltage stimulation (Figure 3c), implying an excellent electrochemical stability of such hydrogels. The electrochemical impedance spectroscopy (EIS) analysis shows that the impedance of 15 wt.% Ti_3_C_2_T_x_-PAA hydrogel is significantly lower than a bare Pt electrode in the frequency range of 1 Hz (Figure 3d), suggesting enhanced ion transportability in the 15 wt.% Ti_3_C_2_T_x_-PAA physical hydrogel. Moreover, over a frequency range of 10^3^~10^5^ Hz (the high-frequency range), impedance plot of the hydrogel exhibits the phase angle (nearly 0°) as well as the value of solution resistance (Rs) that varies from 34.85 to 32.53 Ω. In light of the frequency range of 0.1~10^2^ Hz (the low-frequency line), the 15 wt.% Ti_3_C_2_T_x_-PAA hydrogel represents the electric double layer capacitance (CPE_dl_), membrane resistance (*R*_m_), and charge transfer resistance (*R*_e_) (Figure 3d,e). A reasonable equivalent circuit model of the 15 wt.% Ti_3_C_2_T_x_-PAA hydrogel in PBS solution (pH = 7.4) is also well fitted (Figure 3f).

### 3.4. Adhesion Performance of Ti_3_C_2_T_x_-PAA Hydrogels

**Figure 4:**
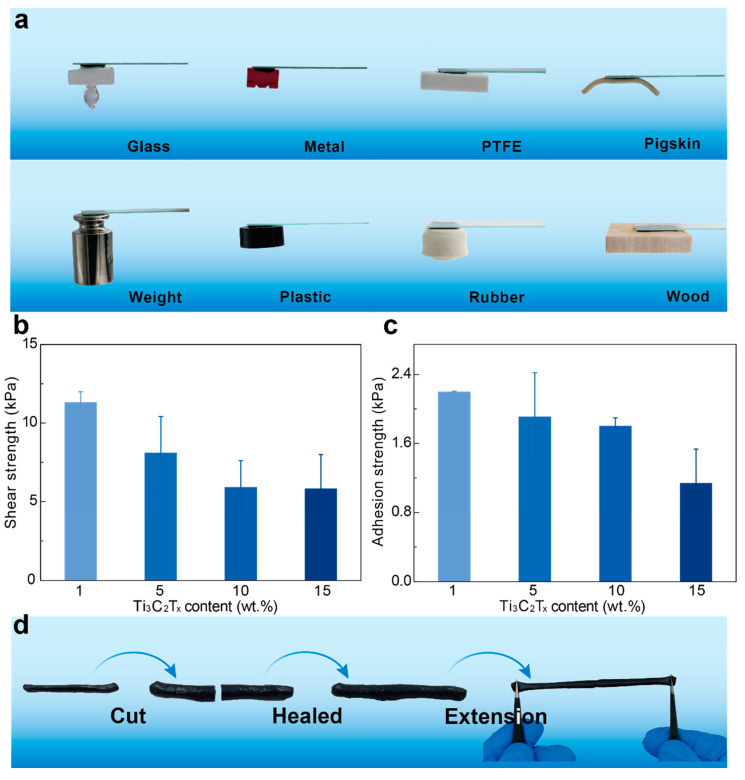
Adhesion property and self-healing property of Ti_3_C_2_T_x_-PAA hydrogels. (**a**) Photographs illustrate the adhesion ability of hydrogels on different substrates (glass, metal, PTFE, weight, rubber, plastic, wood and pigskin). (**b**) Shear adhesion strength of Ti_3_C_2_T_x_-PAA hydrogels on PET substrates. (**c**) Adhesion strength on aluminum substrate. (**d**) Self-healing property of the hydrogel.

To demonstrate the extensive adhesion of hydrogels (Figure 4a), we adhere hydrogels on different substrates, including glass, metal, PTFE, rubber, plastic, wood, and pig skin. Interestingly, the Ti_3_C_2_T_x_-PAA hydrogel can withstand 200 g loading cell when adhering on the substrate. To evaluate the adhesion performance of Ti_3_C_2_T_x_-PAA hydrogels, we adopt standard mechanical tests to measure the adhesion strength on a range of typical substrates (Figure S3). It is found that Ti_3_C_2_T_x_-PAA hydrogels generally exhibit good adhesion strengths on varying substrates (2.13 kPa for glass, 0.44 kPa for metal, 0.42 kPa for PTFE, 0.74 kPa for pigskin, 0.49 kPa for weight, 2.04 kPa for plastic, 0.38 kPa for rubber, and 0.52 kPa for wood).

Additionally, we measure the adhesion strength on aluminum substrate via 90° peeling and adhesion of hydrogels on PET by the lap shear test. In general, the electrostatic force between carboxyl groups on PAA chains and various substrates is the main reason for the adhesion of hydrogels [36]. In our work, the shear strength (5.84 kPa to 11.32 kPa) and adhesion strength (1.14 kPa to 2.20 kPa) of hydrogels show an increasing trend (Figure 4b,c). Due to the increase of the concentration of AA monomers, the electrostatic force between hydrogels and substrates is obviously enhanced, leading to good adhesion of Ti_3_C_2_T_x_-PAA hydrogels against varying substrates.

### 3.5. Self-Healing Properties of Ti_3_C_2_T_x_-PAA Hydrogels

Ti_3_C_2_T_x_-PAA hydrogels are able to self-heal immediately after cutting (Figure 4d). When we cut the hydrogels into two pieces and put them in touch with each other under external force, they heal automatically within several seconds. The self-healed hydrogel could be stretched without obvious decrease in ultimate strain.

### 3.6. Patterning Ti_3_C_2_T_x_-PAA Hydrogels by 3D Printing

Complex patterning/manufacturing techniques have greatly hampered the development of stretchable, adhesive, and conductive hydrogels in various practical applications. Enlightened by the 3D printability of recent MXene materials and conducting polymer ink via controlling the viscosity [4], we developed the 3D printing techniques of Ti_3_C_2_T_x_-PAA hydrogels. The viscosity of Ti_3_C_2_T_x_-PAA inks increased rapidly with increasing Ti_3_C_2_T_x_ contents (Figure S2), rendering good 3D printability of such inks [45,46]. Notably, an excessively high Ti_3_C_2_T_x_ concentration will lead to significant aggregation of hydrogels and nozzle clogging.

**Figure 5:**
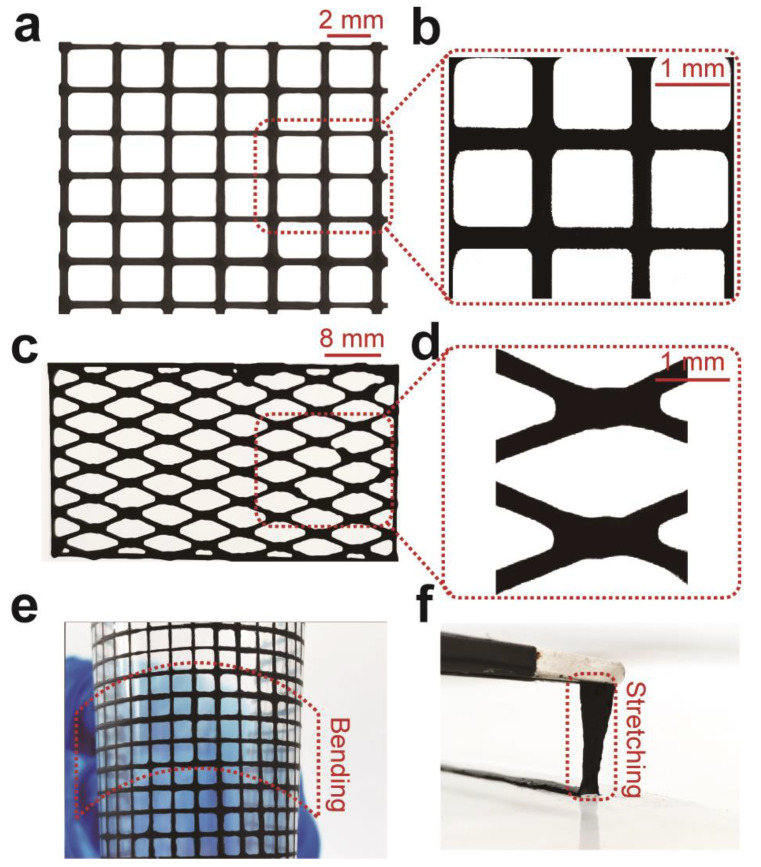
3D printing of Ti_3_C_2_T_x_-PAA hydrogels. (**a**) 3D-printing of a mesh structure and (**b**) its microstructure. (**c**) 3D-printing of a rhombus structure and (**d**) its microstructure. (**e**) Bending of the 3D-printed Ti_3_C_2_T_x_-PAA mesh structure without defect. (**f**) Stretching of the 3D-printed Ti_3_C_2_T_x_-PAA rectangle structure without failure.

To demonstrate the capability of 3D printing such hydrogels, we printed mesh (Figure 5a) and rhombus (Figure 5c) structures with 15 wt.% Ti_3_C_2_T_x_-PAA ink through a 160-µm diameter nozzle onto PET film. The 3D-printed Ti_3_C_2_T_x_-PAA hydrogel pattern displays superior flexibility, good stretchability, and excellent adhesion against the PET substrate (Figure 5e,f), offering a promising platform to fabricate multifunctional materials towards various applications like tissue engineering and neural science [47,48].

## 4. Conclusions

We successfully developed a pre-crosslinking and secondary-crosslinking strategy for the 3D printing of stretchable, adhesive, and conductive Ti_3_C_2_T_x_-PAA hydrogels. The pre-polymerization between Ti_3_C_2_T_x_ nanosheets and AA monomers yields viscous 3D printable inks. Secondary polymerization of 3D-printed patterns by 3D printing realizes a multifunctional Ti_3_C_2_T_x_-PAA hydrogel. The resultant hydrogels are demonstrated to display high stretchability (~622%), high electrical conductivity (5.13 S m^−1^), excellent adhesion (11.32 kPa), and outstanding electrochemical activity and stability. Moreover, the 3D printable Ti_3_C_2_T_x_-PAA inks can be readily printed into various complex patterns like mesh and rhombs with high resolution, which benefits robust integration of wearable and implantable devices. This work not only provides a simple strategy to achieve stretchable, conductive, and self-healable multifunctional hydrogels, but also sets up a 3D printing technique for facile fabrication and integration of diverse bioelectronic devices.

## Data Availability

The raw/processed data required to reproduce these findings cannot be shared at this time due to technical limitations. They are available on request.

## Supplementary Materials

The following supporting information can be downloaded at: https://www.mdpi.com/article/10.3390/polym14101992/s1, Figure S1: Digital images of varying Ti_3_C_2_T_x_-PAA inks after 1 day (a), 2 days (b), and 3 days (c), showing the viscosity increase with time, Figure S2: Rhombus and square patterns for 3D printing of Ti_3_C_2_T_x_-PAA hydrogels. Designing and printing paths for (a) rhombus pattern, (b) square pattern (size: 2 mm × 2 mm), and (c) square pattern (size: 3 mm × 3 mm), Figure S3: Adhesion strength of 1 wt.% Ti_3_C_2_T_x_-PAA hydrogel with varying substrates.; Table S1: Compositions of Ti_3_C_2_T_x_-PAA hydrogels, Table S2: Electrical conductivity comparison of our Ti_3_C_2_T_x_-PAA hydrogels with previously reported conductive hydrogels. References [36,40,41,42,43] are cited in Supplementary Materials.
