# Supplementary material for: 3D Printing of Stretchable, Adhesive and Conductive Ti3C2Tx-Polyacrylic Acid Hydrogels
Source: Polymers (Basel). 2022 May 13;14(10):1992. doi: 10.3390/polym14101992 (PMC9147333; doi:10.3390/polym14101992)
Supplement: Supplementary file 1 [file polymers-14-01992-s001.zip › polymers-1680978-supplementary.pdf]

SUPPLEMENTARY INFORMATION FOR

3D Printing of Stretchable, Adhesive and Conductive $Ti_3C_2T_x$-Polyacrylic Acid Hydrogels

Weijing Zhao[1] †, Jie Cao[2,3] †, Fucheng Wang[2,4] †, Fajuan Tian[2,4], Wenqian Zheng[2,4], Yuqian Bao[1], Kaiyue Zhang[2,4], Zhilin Zhang[2,4], Jiawen Yu[2,4], Jingkun Xu[2], Ximei Liu[2,4*], Baoyang Lu[2,4*]

[1] Department of Endocrinology and Metabolism, Shanghai Jiao Tong University Affiliated Sixth People's Hospital, Shanghai Clinical Center for Diabetes, Shanghai Key Clinical Center for Metabolic Disease, Shanghai Diabetes Institute, Shanghai Key Laboratory of Diabetes Mellitus, Shanghai Jiao Tong University, Shanghai 200240, China; z811wj@163.com (W.Z.); yqbao@sjtu.edu.cn (Y.B.)
[2] Flexible Electronics Innovation Institute, Jiangxi Key Laboratory of Flexible Electronics, Jiangxi Science & Technology Normal University, Nanchang 330013, China; caojie9905@163.com (J.C.); wfc317500626@163.com (F.W.); fajuantian2020@163.com (F.T.); cathy9806@163.com (W.Z.); zky193722@163.com (K.Z.); zhangzhilin1117@163.com (Z.Z.); yujw0629@163.com (J.Y.); xujingkun@tsinghua.org.cn (J.X.)
[3] School of Chemistry & Chemical Engineering, Jiangxi Science & Technology Normal University, Nanchang 330013, China
[4] School of Pharmacy, Jiangxi Science & Technology Normal University, Nanchang 330013, China
* Correspondence: liuxm@jxstnu.edu.cn (X.L.); luby@jxstnu.edu.cn (B.L.) Tel.: +86-7918-8537-967;
† These authors contribute equally to this work.

Table S1. Compositions of $Ti_3C_2T_x$-PAA hydrogels

Ink ratios	$Ti_3C_2T_x$ (g)	AA (g)	H_2O (g)
1 wt.% $Ti_3C_2T_x$-PAA	0.040	3.960	0.531
5 wt.% $Ti_3C_2T_x$-PAA	0.040	0.76	0.531
10 wt.% $Ti_3C_2T_x$-PAA	0.040	0.36	0.531
15 wt.% $Ti_3C_2T_x$-PAA	0.040	0.227	0.531

Table R1 (as Table S2). Electrical conductivity comparison of our $Ti_3C_2T_x$-PAA hydrogels with previously reported conductive hydrogels

Hydrogel sample	Preparation method	Fillers content (wt.%)	Conductivity (S/m)	Refs
$Ti_3C_2T_x$ /PAA	In situ polymerization	0.2	0.42	[36]
$Ti_3C_2T_x$/PAA/Fe^{3+}	In situ polymerization	0.5	3.8	[36]
MXene/PNIPAM/PAAm	Physical crosslinking	1.3	1.092	[40]
MXene/PVA/Zn^{2+}	Freezing-thawing	0.3	0.056	[41]
MXene/PEDOT:PSS	Freezing-thawing	1	1525.8	[42]
$Ti_3C_2T_x$/PAAm/PAAc/Glycerol	In situ polymerization	0.2	1.34	[43]
$Ti_3C_2T_x$ /PAA	In situ polymerization	15	5.13	This work

PNIPAM—poly(N-isopropylacrylamide); PAA—polyacrylic acid; PAAm—polyacrylamide; PVA—poly(vinyl alcohol).

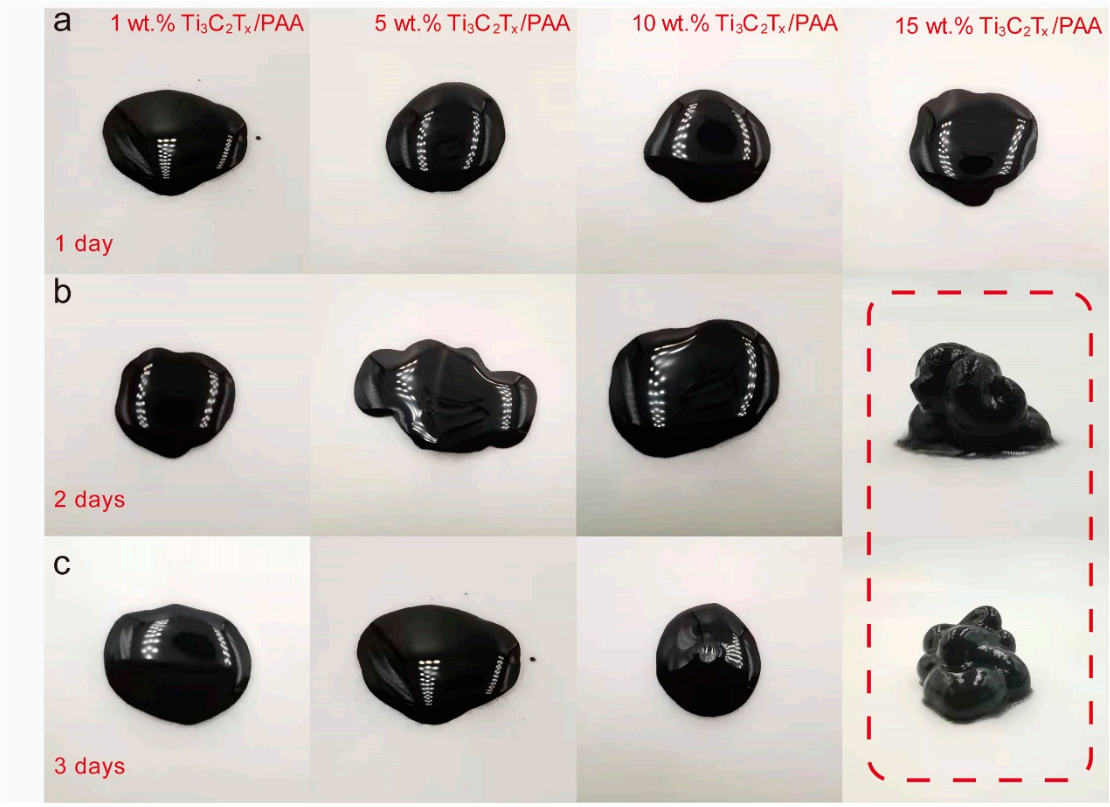

Figure S1. Digital images of varying $Ti_3C_2T_x$-PAA inks after 1 day (a), 2 days (b), and 3 days (c), showing the viscosity increase with time.

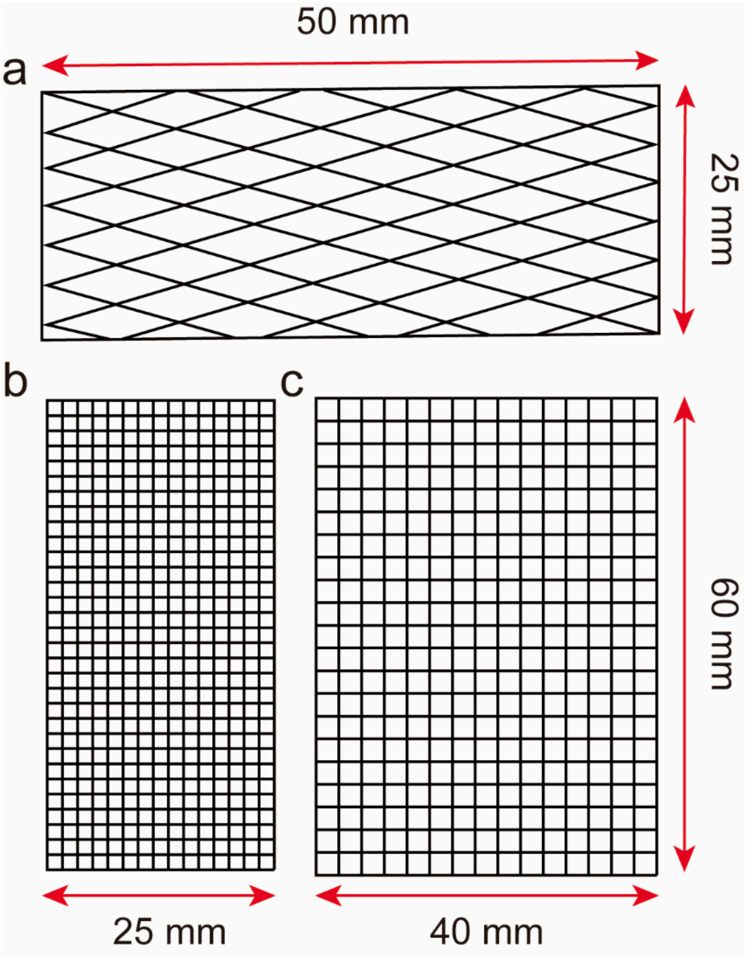

Figure S2. Rhombus and square patterns for 3D printing of $Ti_3C_2T_x$-PAA hydrogels. Designing and printing paths for (a) rhombus pattern, (b) square pattern (size: 2 mm × 2 mm), and (c) square pattern (size: 3 mm × 3 mm).

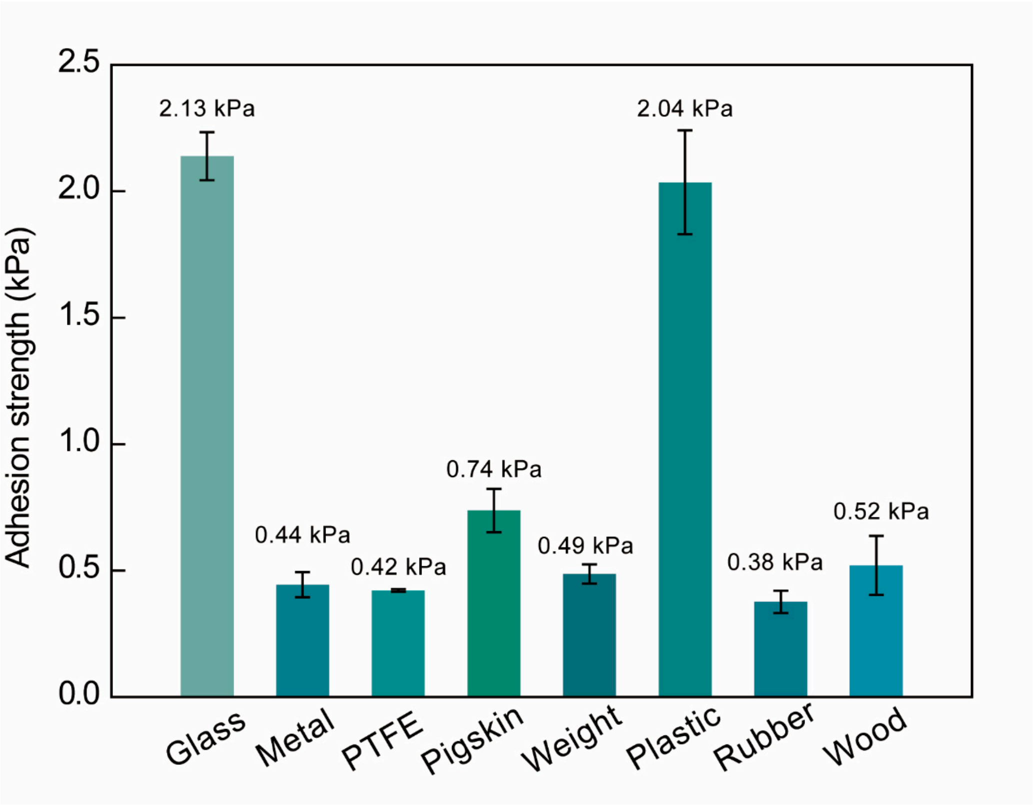

Figure S3. Adhesion strength of 1 wt.% $Ti_3C_2T_x$-PAA hydrogel with varying substrates.
